# Unifying antimicrobial peptide datasets for robust deep learning-based classification

**DOI:** 10.1016/j.dib.2024.110822

**Published:** 2024-08-22

**Authors:** Shuang Peng, Loïc Rajjou

**Affiliations:** Université Paris-Saclay, INRAE, AgroParisTech, Institut Jean-Pierre Bourgin (IJPB), 78000 Versailles, France

**Keywords:** Antimicrobial Peptides (AMPs), Plant, Sequence redundancy, Computational biology, Database analysis

## Abstract

Leguminous crops are vital to sustainable agriculture due to their ability to fix atmospheric nitrogen, improving soil fertility and reducing the need for synthetic fertilizers. Additionally, they are an excellent source of protein for both human consumption and animal feed. Antimicrobial peptides (AMPs), found in various leguminous seeds, exhibit broad-spectrum antimicrobial activity through diverse mechanisms, including interaction with microbial cell membranes and interference with cellular processes, making them valuable for enhancing crop resilience and food safety. In the field of plant sciences, computational biology methods have been instrumental in the discovery and optimization of AMPs. These methods enable rapid exploration of sequence space and the prediction of AMPs using deep learning technologies. Optimizing AMP annotations through computational design offers a strategic approach to enhance efficacy and minimize potential side effects, providing a viable alternative to conventional antimicrobial agents. However, the presence of overlapping sequences across multiple databases poses a challenge for creating a reliable dataset for AMP prediction. To address this, we conducted a comprehensive analysis of sequence redundancy across various AMP databases. These databases encompass a wide range of AMPs from different sources and with specific functions, including both naturally occurring and artificially synthesized AMPs. Our analysis revealed significant overlap, underscoring the need for a non-redundant AMP sequence database. We present the development of a new database that consolidates unique AMP sequences derived from leguminous seeds, aiming to create a more refined dataset for the binary classification and prediction of plant-derived AMPs. This database will support the advancement of sustainable agricultural practices by enhancing the use of plant-based AMPs in agroecology, contributing to improved crop protection and food security.

Specifications Table

**Table:** 

Subject	Biological Sciences / Bioinformatics
Specific subject area	Deep learning modelling of binary sequence classification to predict AntiMicrobial Peptide
Type of data	fasta (amino acid sequence), huggingface dataset.
Data collection	The dataset for the study on antimicrobial peptides (AMPs) was gathered through a rigorous analysis of sequence redundancy across multiple AMP databases, encompassing both naturally occurring and artificially synthesized AMPs. The Dover analyzer (http://mobiosd-hub.com/doveranalyzer/) was used to identify and eliminate redundant AMP entries, thereby unveiling the intricate relationship among 28 distinct AMP databases. To enrich the dataset, non-AMP data was sourced from the UniProtKB database (https://www.uniprot.org/uniprotkb), with a specific focus on plant proteins meeting predetermined criteria regarding sequence length and taxonomy. The non-AMP dataset underwent hierarchical redundancy removal using CD-HIT (https://www.bioinformatics.org/cd-hit/), a widely adopted clustering tool tailored for large sequence databases. Subsequent to data collection, various preprocessing steps were implemented to ensure data integrity and compatibility for downstream analysis. Special characters were systematically removed from the dataset entries to standardize the format and facilitate uniform processing. Furthermore, to distinguish between AMPs and non-AMPs, peptides shared across both datasets were identified and subsequently excluded from the final datasets. The processed AMP and non-AMP datasets were integrated with the Hugging Face dataset module (https://huggingface.co/datasets), a versatile platform catering to the needs of machine learning and data science endeavours. Drawing upon the functionalities offered by the Hugging Face framework ensured efficient integration and facilitated accessibility for subsequent analysis and model development.
Data source location	The data for this study were collected from various publicly available databases, which are typically hosted online and accessible globally (see Table 1). The specific geographical coordinates of the data sources may vary, as they are often maintained by institutions or organizations worldwide. Additionally, the final datasets were integrated and stored using the resources available at our institution, namely the Recherche Data Gouv platform.
Data accessibility	***Please note:****All raw data referred to in this article must be made publicly available in a data repository prior to publication. Please indicate here where your data are hosted (the URL must be working at the time of submission and editors and reviewers must have anonymous access to the repository):* Repository name: … Data identification number: doi:10.1093/bioinformatics/btv180. Direct URL to data: https://entrepot.recherche.data.gouv.fr/dataset.xhtml?persistentId=doi:10.57745/NZ0IRX&version=DRAFT https://huggingface.co/datasets/ps29/amp_dataset_viri https://huggingface.co/datasets/ps29/amp_dataset_faba Instructions for accessing these data: …
Related research article	*None*

## 1 Value of the Data

This section states why these data are of value to the scientific community. Please **provide between 3 and 6 bullet points** and answer at least the questions below (delete the questions afterwards). Each bullet point should be a maximum of 150 words long, and should not include conclusions or inferences:

• The paper describes the creation of a non-redundant Antimicrobial peptides (AMPs) dataset by analyzing sequence overlap across multiple databases, aiming at training robust binary AMP classifier.
• The out-performed AMP classifier accelerated the discovery and optimization of AMPs, which offer a potential solution of the escalating antibiotic resistance issue.
• The model performance is strongly related to the size of training data size. This dataset retrieves the AMPs from the databases in comprehensive way, which could be used as the knowledge base for those researchers who want to develop the specific predictors on their own.
• In addition, despite the large number of available models, the number of standardized datasets is still very limited. This dataset is expected to be one of the standardized datasets in the future.
• Finally, in the context of deep learning modeling, the more standardized Hugging Face release makes it easier to access and use the dataset.

## 2 Background

The excessive use of synthetic chemical pesticides has led to environmental and health concerns, including the development of resistance among various harmful agricultural pests and pathogens [1]. The search for sustainable and effective biotic control options is a significant focus of contemporary agricultural research [2].

Antimicrobial peptides (AMPs), especially those extracted from plant resources, offer a promising avenue for developing new, environmentally friendly pest control solutions. These peptides are an important part of the innate immune system of various organisms [3], with broad activity against microorganisms by binding, penetrating, and interfering with microbial cells [4,5].

The in-silico approach enhances the design accuracy and discovery of potential AMPs through pattern recognition, sequence alignment, and machine learning [6,7]. This method reduces time and costs by efficiently exploring a sequence space for antimicrobial activity prediction.

The impact of dataset size on predictive model performance is critical, and increasing AMP databases play a vital role as the AMP repository. However, many AMP databases have overlapping sequences for various purposes [8]. This study aims to analyze sequence redundancy in different AMP databases and create a non-redundant sequence database for AMP binary classification.

## 3 Data Description

Two AMP classification datasets (*Fabaceae* and *Viridiplantae*) were organized according to the non-amp sources, where the training set of *Fabaceae* contained 58,119 entries, the validation set contained 18,231 entries, and the test set contained 14,530 entries, and the training set of *Viridiplantae* contained 91,131 entries, the validation set contains 28,548 entries, and the test set contains 22,783 entries.

Each of the two datasets corresponds to a HuggingFace Dataset, and each entry contains the five features (index, id, sequence, length and label).

## 4 Experimental Design, Materials and Methods

### 4.1 Data Source

The 28 databases used to construct the AMP dataset are described in Table 1. There were 22 AMP datasets in [1] used AMP data source, with APD database updated [3] and the other 21 unchanged. In addition, 6 new databases (BaAMPs, dbAMP, DRAMP General, DRAMP Patent, DRAMP Clinical, DRAMP Specific) have been added.

**Table 1:** Antimicrobial peptide database as input to amp-dover, including a description of entry source, target, current availability, last update date, total number of entries and references.

Databases	Type	Focused on	Available	Last update	Entries	Reference
Peptaibol	Specific	Peptaibol family	Absent	2004	316	10.1093/nar/gkh077
Defensins	Specific	Defensin family of AMPs	Absent	2007	536	10.1093/nar/gkl866
AVPdb	Specific	Antiviral peptides	Present	2013	2,059	10.1093/nar/gkt1191
MilkAMP	Specific	AMPs of dairy origin	Present	2013	385	10.1007/s13594-013-0153-2
AMSDb	Specific	Eukaryotic AMPs	Absent	01/11/2004	893	10.2174/1381612023395475
AMPer	Specific	Eukaryotic AMPs	Present	01/02/2007	988	10.1093/bioinformatics/btm068
PenBase	Specific	Penaeidin family of AMPs	Absent	01/07/2008	28	10.1016/j.dci.2005.04.003
RAPD	Specific	Recombinantly produced AMPs	Absent	01/03/2010	179	10.1111/j.1574-6968.2008.01357.x
DAMPD	General	General AMPs	Absent	01/09/2011	1,232	10.1093/nar/gkr1063
PhytAMP	Specific	AMPs from plants	Present	01/01/2012	273	10.1093/nar/gkn655
DADP	Specific	Anuran defense peptides	Absent	01/03/2012	2,571	10.1093/bioinformatics/bts141
Bagel I	Specific	Bacteriocins	Absent	01/01/2013	158	10.1093/nar/gkq365
Bagel II	Specific	Bacteriocins	Absent	01/01/2013	228	10.1093/nar/gkq365
Bagel III	Specific	Bacteriocins	Absent	01/01/2013	93	10.1093/nar/gkq365
LAMP Experimental	General	General AMPs	Present	01/03/2013	3,191	10.1371/journal.pone.0066557
LAMP Patent	Specific	Patented AMPs	Present	01/03/2013	1,491	10.1371/journal.pone.0066557
YADAMP	General	General AMPs	Absent	01/03/2013	2,525	10.1016/j.ijantimicag.2011.12.003
Bactibase	Specific	Bacteriocins	Present	01/10/2014	227	10.1186/1471-2180-10-22
BaAMPs	Specific	Against microbial biofilms	Absent	13/09/2019	221	10.1186/1471-2180-10-22
dbAMP	General	General	Present	01/06/2021	18,345	10.1093/nar/gkab1080
APD3	General	General AMPs	Present	01/08/2021	3,273	10.1093/nar/gkv1278
CAMP Patent	Specific	Patented AMPs	Absent	01/11/2013	1,716	10.1093/nar/gkv1051
CAMP Structure	General	3D structures of AMPs	Absent	01/11/2013	682	10.1093/nar/gkv1051
CAMP Validated	General	General AMPs	Absent	01/11/2013	2,602	10.1093/nar/gkv1051
DRAMP General	General	General	Present	04/07/2023	6,034	10.1093/nar/gkab651
DRAMP Patent	Specific	Patented AMPs	Present	04/07/2023	16,110	10.1093/nar/gkab651
DRAMP Clinical	Specific	Clinical AMPs	Present	04/07/2023	40	10.1093/nar/gkab651
DRAMP Specific	Specific	Specific AMPs	Present	04/07/2023	6,097	10.1093/nar/gkab651

The non-AMPs were retrieved from the UniProtKB database. Catering to the research purpose of predicting AMPs from plant resources, especially from legume seeds, the protein selection was limited to the plant kingdom. Database searches were conducted according to the following criteria:

• length: from 5 to 155;
• taxonomy_id: taxonomic levels, contains *Fabaceae* [3803], *Viridiplantae* [33090];
• keyword: Gene Ontology Annotation (Molecular Function), eliminates Antimicrobial [KW-0929], Pathogenesis-related protein [KW-0568], Antiviral protein [KW-0930]

Two non-AMP data sets, *Fabaceae* and *Viridiplantae*, were generated, with 59,606 and 682,607 entries respectively.

### 4.2 Data Preprocessing

Dover analyzer was used to remove redundant AMPs. The results revealed the relationship between the 28 AMP databases. The percentage of non-redundant amp content in all databases is shown Table 2, which shows that all AMP databases contain more than 50% non-redundant AMP content. Two heatmaps show the percentage of overlap between different databases, where Table 2 represents the percentage of row databases contained in the column databases, while Table 2 represents the percentage of row databases that are completely stored in each number of databases. A total of 44,099 AMP entries were obtained after preprocessing.

**Table 2:** The total number of sequences before/after filtration and unique sequences percentage in each database.

Database	Total	Non-duplicate	Percent
MilkAMP_clean	385	313	81,3
DRAMP_Clinical_clean	34	28	82,35
Bagel_I_clean	158	154	97,47
DRAMP_Specific_clean	6097	6044	99,13
YADAMP_clean	2525	2525	100
DRAMP_Patent_clean	16104	14212	88,25
PenBase_clean	28	28	100
RAPD_clean	179	122	68,16
AVPdb_clean	2059	1817	88,25
AMSDb_clean	893	858	96,08
LAMP_Patent_clean	1491	1491	100
DRAMP_General_clean	6027	5823	96,62
Bagel_III_clean	93	71	76,34
APD_clean	3273	3238	98,93
AMPer_clean	988	948	95,95
Bactibase_clean	227	215	94,71
DAMPD_clean	1232	1199	97,32
CAMP_Structure_clean	929	708	76,21
DADP_clean	2571	2460	95,68
CAMP_Patent_clean	1716	1675	97,61
Peptaibol_clean	316	198	62,66
LAMP_Experimental_clean	3191	3190	99,97
Defensins_clean	536	507	94,59
dbAMP_clean	18345	18312	99,82
Bagel_II_clean	228	217	95,18
CAMP_Validated_clean	16640	15507	93,19
BaAMPs_clean	212	188	88,68
PhytAMP_clean	273	272	99,63

The redundant non-AMPs in *Viridiplantae* dataset were hierarchically removed by CD-HIT (Table 2).

All special characters except common amino acids in AMP and non-AMP were removed through preprocessing. Finally, the peptides shared between AMP and non-AMP were deleted.

### 4.3 Dataset Preparation

The AMPs and non-AMPs (including *Fabaceae* and *Viridiplantae*) were used to build the dataset for amp prediction modeling. Where there were 8,876 amps from 11 validated databases selected for test data (Table 3) and the remaining 35,229 amps used for training data (Table 4).

**Table 3:** Results of hierarchical protein clustering on the *Viridiplantae* dataset using cd-hit, the first column shows the similarity metric for each clustering hierarchy, and the last two columns correspond to the input and output corresponding protein sequence entries.

Clustering percentage	Input entries	Output entries
80,00 %	442,923	365,347
70,00 %	365,347	344,001
60,00 %	344,001	304,472
50,00 %	304,472	224,918
40,00 %	224,918	98,859
30,00 %	98,859	98,389
20,00 %	98,389	98,357
10,00 %	98,357	98,357
5,00 %	98,357	98,357
1,00 %	98,357	98,357

**Table 4:** AMP database selection for the test set, containing input and output entries and the percentage of non-redundant peptides to itself.

Output	Input	Percentage of non-redundant peptides
From 40 Exported	34	protein sequences to DRAMP_Clinical_clean(85%)
From 273 Exported	273	protein sequences to PhytAMP_clean(100%)
From 536 Exported	536	protein sequences to Defensins_clean(100%)
From 929 Exported	929	protein sequences to CAMP_Structure_clean(100%)
From 1232 Exported	1232	protein sequences to DAMPD_clean(100%)
From 1491 Exported	1491	protein sequences to LAMP_Patent_clean(100%)
From 1716 Exported	1716	protein sequences to CAMP_Patent_clean(100%)
From 2525 Exported	2525	protein sequences to YADAMP_clean(100%)
From 2571 Exported	2571	protein sequences to DADP_clean(100%)
From 3191 Exported	3191	protein sequences to LAMP_Experimental_clean(100%)
From 3273 Exported	3273	protein sequences to APD_clean(100%)

The *Fabaceae* and *Viridiplantae* dataset were divided into training set and test set by equidistant sampling, containing 78,685 / 19,672 and 37,420 / 9,355 non-AMPs respectively.

The dataset was then integrated through HuggingFace dataset module.

## Limitations

**Fig. 1:**
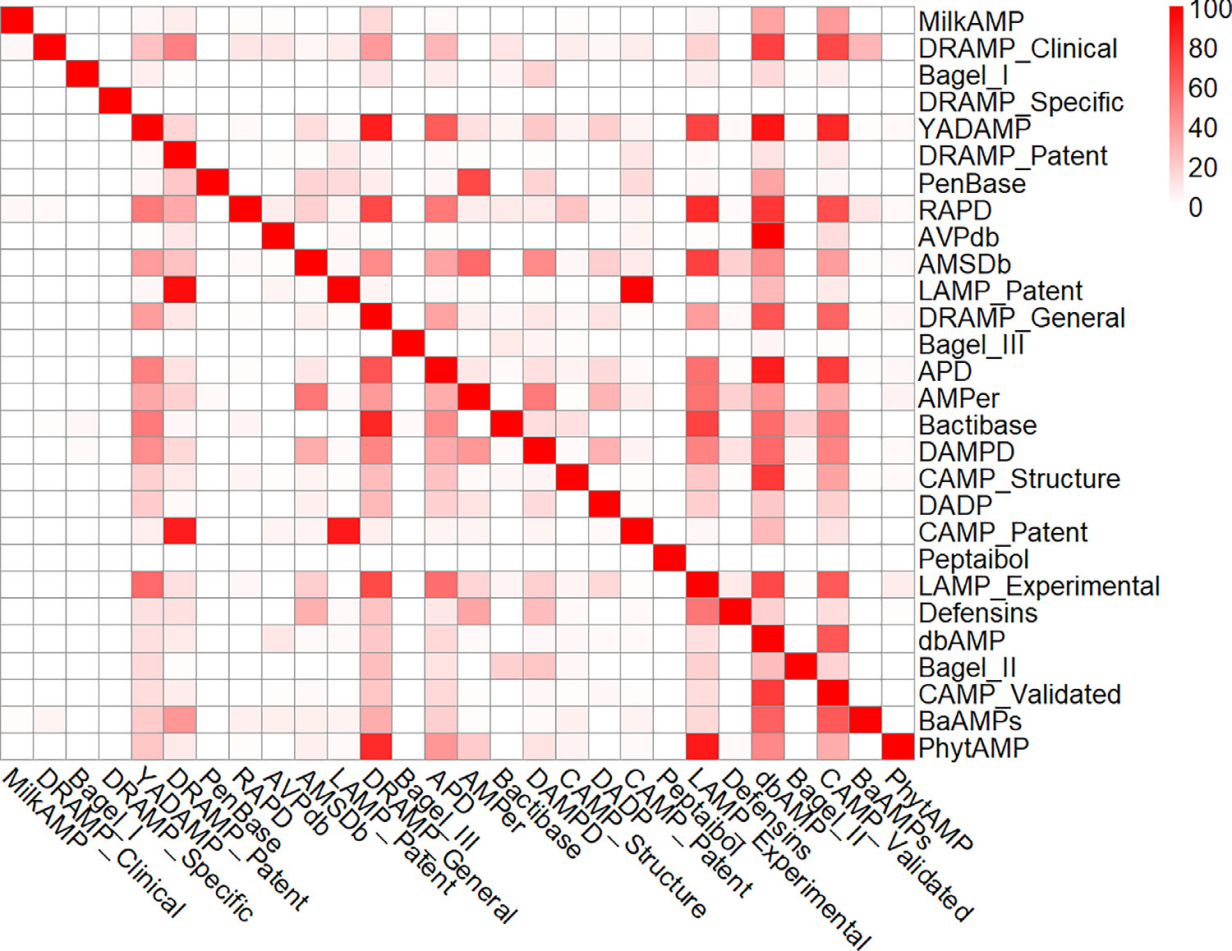
Heatmap of sequence overlap between each two AMP databases. The overlaps were represented by the percentage of overlap sequence in database of row.

While the antimicrobial peptide dataset presented in this research offers a quantitative edge, it lacks a comprehensive depiction of the data label, including details like origin, specific antimicrobial targets, immune test parameters, and whether it is synthetically produced or predicted. It is recommended that future users of the dataset supplement it with more elaborate descriptions to enhance the efficacy of predictive model (Fig. 1 and Table 5).

**Table 5:** AMP database selection for the train/validation set, containing input and output entries and the percentage of non-redundant peptides to itself.

Output	Input	Percentage of non-redundant peptides
From 28 Exported	28	protein sequences to PenBase_clean(100%)
From 93 Exported	93	protein sequences to Bagel_III_clean(100%)
From 158 Exported	158	protein sequences to Bagel_I_clean(100%)
From 179 Exported	179	protein sequences to RAPD_clean(100%)
From 221 Exported	212	protein sequences to BaAMPs_clean(95%)
From 227 Exported	227	protein sequences to Bactibase_clean(100%)
From 228 Exported	228	protein sequences to Bagel_II_clean(100%)
From 316 Exported	316	protein sequences to Peptaibol_clean(100%)
From 385 Exported	385	protein sequences to MilkAMP_clean(100%)
From 893 Exported	893	protein sequences to AMSDb_clean(100%)
From 988 Exported	988	protein sequences to AMPer_clean(100%)
From 2059 Exported	2059	protein sequences to AVPdb_clean(100%)
From 6034 Exported	6029	protein sequences to DRAMP_General_clean(99%)
From 6097 Exported	6097	protein sequences to DRAMP_Specific_clean(100%)
From 16110 Exported	16110	protein sequences to DRAMP_Patent_clean(100%)
From 16640 Exported	16640	protein sequences to CAMP_Validated_clean(100%)
From 18345 Exported	18345	protein sequences to dbAMP_clean(100%)

## Ethics Statement

The current work does not involve human subjects, animal experiments, or any data collected from social media platforms. Therefore, we confirm that our research strictly adheres to the guidelines for authors provided by Data in Brief in terms of ethical considerations.

## CRediT authorship contribution statement

**Shuang Peng:** Conceptualization, Investigation, Methodology, Visualization, Data curation, Writing – original draft. **Loïc Rajjou:** Conceptualization, Investigation, Supervision, Validation, Writing – review & editing.

## Data Availability

amp_dataset_viri (Original data) (Hugging Face). amp_dataset_faba (Original data) (Hugging Face). Unifying Antimicrobial Peptide Datasets for Robust Deep Learning-Based Classification (Original data) (Recherche Data Gouv).
